# Neural correlates of gait adaptation in younger and older adults

**DOI:** 10.1038/s41598-023-30766-x

**Published:** 2023-03-08

**Authors:** Tyler Fettrow, Kathleen Hupfeld, Chris Hass, Ofer Pasternak, Rachael Seidler

**Affiliations:** 1 Department of Applied Physiology and Kinesiology, University of Florida, Gainesville, FL 32605 USA; 2 Department of Psychiatry, Brigham and Women’s Hospital, Harvard Medical School, Boston, MA USA; 3 Present Address: NASA Langley Research Center, Hampton, VA USA

**Keywords:** Ageing, Neurophysiology, Neural ageing

## Abstract

Mobility decline is a major concern for older adults. A key component of maintaining mobility with advancing age is the ability to learn and adapt to the environment. The split-belt treadmill paradigm is an experimental protocol that tests the ability to adapt to a dynamic environment. Here we examined the magnetic resonance imaging (MRI) derived structural neural correlates of individual differences in adaptation to split-belt walking for younger and older adults. We have previously shown that younger adults adopt an asymmetric walking pattern during split-belt walking, particularly in the medial-lateral (ML) direction, but older adults do not. We collected *T*$_1$-weighted and diffusion-weighted MRI scans to quantify brain morphological characteristics (i.e. in the gray matter and white matter) on these same participants. We investigated two distinct questions: (1) Are there structural brain metrics that are associated with the ability to adopt asymmetry during split-belt walking; and (2) Are there different brain-behavior relationships for younger and older adults? Given the growing evidence that indicates the brain has a critical role in the maintenance of gait and balance, we hypothesized that brain areas commonly associated with locomotion (i.e. basal ganglia, sensorimotor cortex, cerebellum) would be associated with ML asymmetry and that older adults would show more associations between split-belt walking and prefrontal brain areas. We identified multiple brain-behavior associations. More gray matter volume in the superior frontal gyrus and cerebellar lobules VIIB and VIII, more sulcal depth in the insula, more gyrification in the pre/post central gyri, and more fractional anisotropy in the corticospinal tract and inferior longitudinal fasciculus corresponded to more gait asymmetry. These associations did not differ between younger and older adults. This work progresses our understanding of how brain structure is associated with balance during walking, particularly during adaptation.

## Introduction

Mobility decline is a major concern for older adults. Many components of the human sensorimotor system degrade with age, which can shift the neural controls of mobility from being more automated (spinal cord, subcortical), to more cognitive (cortical)^1^. Understanding how the neural correlates of mobility change with age will help us better understand the degradation of mobility with age. A key component of mobility decline with age is a decrease in adaptability; decreased sensorimotor adaptability has been shown for older adults in both the upper limbs^2,3^ and in lower limbs locomotor tasks^4^. The split-belt treadmill paradigm, in which a treadmill belt under each foot moves at a different speed, forces individuals to adopt a new gait pattern^5^. This adaptation can be characterized by quantifying how quickly people adapt and through changes in which gait parameters^6–9^. Here we focused on identifying brain structural correlates of gait adaptation properties reflected by using the common split-belt treadmill paradigm.

Different regions of the brain are thought to contribute to different aspects of mobility^10^, and these contributions may change with age^1,11^. These findings have mostly been reported in the context of normal over ground walking. For example, Rosano et al reported that smaller sensorimotor and frontoparietal gray matter volumes were associated with shorter step length and longer double support times in older adults^12^. Additionally, Rosso et al found that higher step length variability related to less gray matter in the anterior cingulate and hippocampus, in older adults^13^. Hippocampal volume has been a common metric that shows an association with mobility (more volume associated with better performance)^14,15^. White matter microstructure (fractional anistropy), regardless of age, has been shown to associate with better gait performance (increased step length and less variability)^16^ as well as improved safety margin^17^, and may also be a factor in inefficient brain function during locomotion (i.e. activity in non-task relevant brain areas)^18^. Cortical thickness also shows associations with walking performance^19,20^, but studies that investigate cortical surface measurements in relation to mobility are limited.

We recently investigated group differences in cortical surface measurements and their relationship with dual task walking performance^20^. In concordance with previous reports^21,22^, older adults presented with reduced gray and white matter relative to younger adults, particularly within sensorimotor processing areas. The older adults showed a relationship of lower cortical thickness in temporal cortex, shallower sulcal depth in the frontal, sensorimotor, and parietal cortices, larger ventricular volume, and lower axial and greater radial superior longitudinal fasciculus diffusivity (adjusted for free water) with greater dual task cost during walking. In the current paper we examine on the same cohort whether these structural brain metrics are associated with split-belt walking.

Previous research with patients with brain damage or movement disorders has shed light on the neural correlates of split-belt treadmill adaptation. People with Parkinson’s disease, which largely impacts dopaminergic transmission, do not adapt their step length to the same degree as age matched controls^23^. People with cerebellar dysfunction exhibit a reduced ability to learn and retain adaptations such as step length asymmetry^24^. Moreover, transcranial magnetic stimulation has been used to show that locomotion adaptation is associated with cerebellar function^25^. Cerebellar transcranial direct current stimulation (tDCS) has been used in an effort to accelerate locomotor adaptation, but with mixed results. Specifically cerebellar tDCS has been shown to slow readaptation in one study^26^, result in no differences in readaptation between the sham and stimulation group in another study^27^, and to produce differing effects in initial adaptation performance based on the polarity of the stimulation^27^. Additionally, a recent randomized, double-blind tDCS study that suppressed the left posterior parietal cortex increased the number of steps to adapt during the initial adaptation of split-belt treadmill walking, and increased the magnitude of after effects^28^. The conflicting results of the few studies that have investigated the effects of brain stimulation on split-belt treadmill adaptation may be due to individual variability in stimulation responsiveness^29^. Moreover, these previous studies have focused on step length and time as indices of adaptation. It is unclear whether the neural correlates of ML adaptive changes would differ.

In a recent publication we reported that anterior-posterior (AP) and ML gait parameters show different adaptation effects in response to the split-belt treadmill paradigm, in addition to age differences in ML effects of split-belt walking^9^. Previous research supports the notion that different aspects of locomotion have different neural controls. That is, ML gait parameters likely require higher level neural control^30–32^ whereas AP gait parameters may result primarily from passive dynamics. This suggests that a gait parameter such as step length may be more passively controlled compared to a gait parameter such as step width. In our recent publication^9^, we showed that younger adults adopted an asymmetry in ML gait parameters during the split condition (belts/feet moving at different speeds), whereas older adults maintained symmetry. This finding suggests that the younger adults are behaving differently for a benefit, which in this case may be lower metabolic expenditure. These findings in combination with existing literature leads us to address two questions here: (1) Are there structural brain metrics that are associated with the ability to adopt asymmetry during split-belt walking; and (2) Are there different brain-behavior relationships for younger and older adults? We hypothesized that brain areas commonly associated with locomotion (i.e. basal ganglia, sensorimotor cortex, cerebellum) would be associated with ML asymmetry, but not step length asymmetry due to its hypothesized mostly passive control. Furthermore, given the extensive literature showing more prefrontal cortical engagement in older adults^1,11^, we expected older adults to have brain-behavior relationships in different brain areas than younger adults. That is, given that older adults show activity in more of the brain during walking than young adults, their split-belt walking adaptation performance may correlate with brain structural metrics more expansively than seen in young adults.

## Methods

### Participants

We analyzed data from 31 younger adults (17 female; 22.3±3.9 years) and 19 older adults (10 female; 72.0±5.0) from the data set described in^20,33^. Table 1 displays additional demographic information of the participants. Participants were recruited through the distribution of flyers across the greater north-central Florida area including at senior centers and retirement communities, advertisements via the University of Florida healthcare system, and word of mouth. This experiment was approved by the University of Florida Institutional Review Board. All participants provided written informed consent to participate.

**Table 1:** Demographics and gait asymmetry scores stratified by age group.

	YA	OA
Demographics		
Age	22.3±3.9	72.0±5.0
Sex	17/31 F	10/19 F
BMI	22.2±2.7	26.3±2.6
Leg length (mm)	911±50.0	887±52.5
Gait Parameters		
$\Delta$ CoM (%)	−4.6±5.2	−1.7±6.0
$\Delta$ $\int$CoP-CoM (%)	−63.3±68.1	11.4±51.9
$\Delta$ Step-CoM (%)	29.0±16.0	3.2±23.4
$\Delta$ Step Length (%)	11.7±8.0	16.6±15.0

The values are displayed as mean ± standard deviation. The gait asymmetry scores, reflect the $\Delta$ magnitude of asymmetry of the respective gait parameter at the plateau of adaptation during the split condition of the split belt treadmill paradigm. See sections “Split-belt adaptation assessments” and “Quantifying Split-belt Outcome Variables” for details. YA = younger adults and OA = older adults.

### Ethics approval and consent to participate

Subjects provided informed verbal and written consent to participate. Written informed consent was obtained from the individual for the publication of any potentially identifiable images or data included in this article. The experiment was approved by the University of Florida Institutional Review Board (IRB ID: IRB201801417). All methods were carried out in accordance with relevant guidelines and regulations.

### Consent for publication

All authors provided approval for publication.

### Split-belt adaptation assessments

We administered a treadmill warm-up lasting 5 min at participants’ self-selected speed to allow them to accommodate to walking on the treadmill. For the remainder of the walking trials, the belts moved at a fixed speed for all participants. We then stopped the treadmill before beginning the baseline walking trials, consisting of a *baseline slow* (0.7m/s), *baseline fast* (1.4m/s), and another *baseline slow* (0.7m/s) walking trial, each lasting 2 min. These trials acclimated the participants to the slow and fast walking speeds. Then we began the *split* trial, where the left belt was moving at the fast speed, and the right belt was moving at the slow speed, lasting 10 min. The *split* trial was followed by an *after* adaptation trial, where both belts were fixed at the slow speed to identify any aftereffects. Participants also performed a *readaptation* and a *washout* trial. The methods for this study were already described in detail in our previous publication^9^.

### Structural images acquisition and processing

Participants completed MRI scans in a Siemens MAGNETOM Prisma 3T scanner (Siemens Healthcare, Erlangen, Germany) with a 64-channel head coil. Structural image acquisitions included a 3D *T*$_1$-weighted anatomical image (repetition time (TR) = 2000 ms, echo time (TE) = 3.06 ms, flip angle = 8$^\circ$, field of view = 256 $\times$ 256 mm$^2$, slice thickness = 0.8 mm, 208 slices, voxel size = 0.8 mm$^3$) and a diffusion-weighted spin-echo prepared echo-planar image (5 *b*$_0$ scans (without diffusion weighting), 64 gradient directions with diffusion weighting 1000 s/mm$^2$, TR = 6400 ms, TE = 58 ms, isotropic resolution = 2 $\times$ 2 $\times$ 2 mm, FOV = 256 $\times$ 256 mm$^2$, 69 slices, phase encoding direction = anterior to posterior). An additional set of 5 *b*$_0$ scans with phased encoding in the opposite direction (posterior to anterior) were collected to correct for distortions in the magnetic field.

Voxelwise analyses were performed on the following brain maps: (1) cerebrum gray matter, (2) cerebellum volume, (3) cortical thickness, (4) sulcal depth, (5) gyrification, (6) fractal dimension, and (7) white matter fractional anisotropy (free-water corrected).

The cerebrum gray matter, cortical thickness, sulcal depth, gyrification, and fractal dimension metrics were derived from the *T*$_1$-weighted anatomical image via CAT12 (version r1725;^34^). Gyrification and fractal dimension are measures of cortical complexity^35,36^. CAT12 is an automated *T*$_1$ processing pipeline that allows for visualization of intermediate processing steps for quality assurance purposes. The resulting cerebrum gray matter image maps were smoothed with an 8mm full width at half maximum kernel. Additionally, all gray matter volume results are reported as image intensity (raw probability values). Gray matter intensity is directly proportional to gray matter volume; larger intensity values indicate higher volume^34^. We smoothed the cortical thickness map with a 15mm kernel and the sulcal depth, gyrification, and fractal dimension maps with a 20mm kernel (recommended by^34^).

We applied a separate processing stream to the cerebellum using the CEREbellum Segmentation (CERES) pipeline^37^ and the Advanced Normalization Tools package (ANTs; v1.9.17;^38,39^). CERES is an automated, cloud-based software that extracts the cerebellum from the raw *T*$_1$-weighted anatomical image. We then used ANTs (rigid, affine, and SyN transforms) to warp the extracted cerebellum to the spatially unbiased infratentorial (SUIT) template^40,41^. Next we calculated the Jacobian determinant (modulated image) using CreateJacobianDeterminantImage within ANTs software suite, mimicking the processing steps we performed in CAT12, and multiplied this image to the transformed image to preserve the raw tissue values. Finally, we smoothed the resulting cerebellar volume image with a 2 mm kernel. We examined cerebellar total volumes (white and gray matter) in this analysis due to difficulties associated with separation of cerebellar gray and white matter. The voxel intensity values therefore have a greater range compared to the cerebrum gray matter.

Fractional anisotropy was derived from the diffusion-weighted image via a combination of ExploreDTI graphical toolbox (v4.8.6; www.exploredti.com;^42^), the FMRIB Software Library (FSL; v6.0.1;^43–46^), and custom free-water correction performed in MATLAB^47^. First, we performed a signal drift correction on the raw diffusion-weighted data using ExploreDTI^48^. Then we used FSL’s *topup* function to estimate the susceptibility-induced off-resonance field^45^). We then used the *eddycuda* function within FSL to correct for eddy current distortions, EPI distortions, and head movements^46^.

Next, we estimated the free-water fractional volume and free-water corrected fractional anisotropy using custom MATLAB code^47^. We used the non free-water corrected fractional anisotropy maps with the tract-based spatial statistics (TBSS) pipeline^49^ within FSL, to generate a mean fractional anistropy skeleton in common space (i.e., the FMRIB58FA 1mm isotropic template). The free-water corrected fractional anisotropy images were projected onto the mean fractional anisotropy skeleton using nonlinear registration^50^. This procedure resulted in a skeletonised free-water corrected fractional anisotropy map, which we used for the current brain-behavior associations analysis.

### Quantifying split-belt outcome variables

The methods involving the analysis of this behavioral data have been published previously^9^. Here, we focus on four spatiotemporal gait parameters that inform us about balance adaption during the split-belt paradigm. These four parameters are described below in the Outcome Variables section. All of the data are represented as a change in response from the average of the baseline slow steps ($\Delta$). We use the following definitions to quantify the gait parameters at every step:

Outcome Variables

- CoM: ML position of the middle of the posterior hip markers at heel strike of the swing foot.
- $\int$CoP-CoM:Displacement between the CoM and the CoP from the slow baseline steps, integrated over the single stance phase.
- Step-CoM: Displacement of the swing foot heel marker from the CoM at heel-strike.
- Step Length: Distance (in the AP direction) from the trailing foot to the leading foot heel marker at the leading foot heel-strike.

We calculated the percent change from the slow condition ($\Delta$) for each variable, on each foot, then calculated the symmetry of the ($\Delta$) between feet. Following^7^, we quantified the rate of adaptation based on the number of steps it took for a variable to reach plateau. The plateau was defined as the average value during the last 50 steps, and the threshold for reaching plateau was defined as the step when the next 9 consecutive steps remained within 2 standard deviations of the plateau. We calculated the magnitude of symmetry change at the plateau for each participant and all gait parameters for the split condition. This magnitude at plateau of symmetry score was then inserted into the SPM model (discussed below) to identify brain-behavior correlations. Due to limited marker supply, we used the middle of the two posterior hip markers as a proxy for the body CoM position^51^. Here we only consider the symmetry score during the split condition since this is where we previously identified the majority of the group differences^9^.

### Brain behavior correlations

We tested two types of statistical models to address our two questions, for each brain metric map (i.e. 7 maps in total): (1) Are there structural brain metrics that are associated with the ability to adopt asymmetry during the split-belt walking; and (2) Are there different brain-behavior relationships for younger and older adults? All models consisted of linear models with covariates constructed in SPM12 (estimated in CAT12 for surface metrics). Therefore we had a total of 14 separate models (7 brain metric maps x 2 questions) in which we tested for a correlation between each brain metric map and the gait parameters. The distinction between models for questions 1 and 2 was in the organization of the covariates of interest (i.e. the symmetry scores). For question 1, we tested for clusters in the brain maps that shared similar correlations across both age groups, and for question 2, we were interested in the interaction of age group and the gait parameter being tested. The symmetry scores included three gait parameters that inform us about ML balance control including (1) $\Delta$ CoM, (2) $\Delta$ Step-CoM, and (3) $\Delta \int$ CoM-CoP. All symmetry scores were included in each of the models where the brain metric was the dependent variable. Age group and sex were used as covariates. Total intracranial volume was also used as a covariate for models that involved cerebrum gray matter and cerebellum volume^34^. We re-estimated all models with the Threshold-Free Cluster Enhancement (TFCE; http://dbm.neuro.uni-650jena.de/tfce) toolbox using the default 5,000 permutations^52^. For each model, statistical significance was established at p<.05, after family-wise error (FWE) correction. Since we included all the gait metrics in each brain metric model, multiple comparisons across the gait metrics are accounted for, and the family-wise error correction corrects for multiple comparisons across brain voxels. Significant brain clusters were anatomically identified based on the appropriate atlas for that brain metric: the Automatic Anatomical Labeling (AAL3) atlas^53^ for cerebrum gray matter, SUIT^40,41^ for cerebellum volume, DK40^54^ for surface measures (cortical thickness, sulcal depth, fractal dimension, and gyrification), and JHU-ICBM tract atlas^55,56^ for fractional anisotropy.

## Results

### Associations between cerebrum gray matter/cerebellum volume, and asymmetry

**Figure 1:**
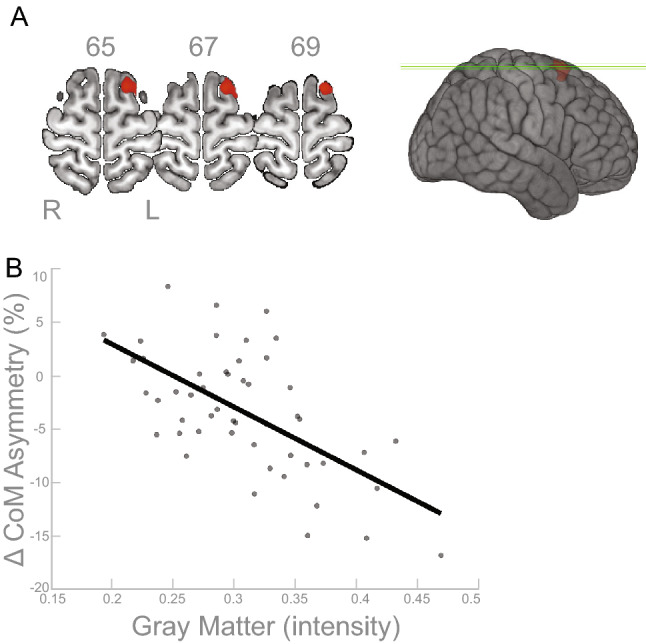
Cerebrum Gray Matter and CoM Results. (**A**) Results of the FWE corrected t-test looking for clusters in the cerebrum gray matter volume that are associated with asymmetry in the CoM during the split condition of the split-belt treadmill paradigm. The highlighted area corresponds to the cluster/s that passed the *p*$_{FWE-corr}<$ 0.05 threshold. According to the AAL3 template, the peak of the significant cluster is located in the left superior frontal gyrus. (**B**) For illustrative purposes only, the linear relationship of the peak result coordinate and asymmetry score are displayed (higher intensity indicates higher volume). The asymmetry score of the gait parameter is calculated as the difference of the variable between slow stance phase and fast stance phase % change from baseline slow condition ($\Delta$). Any deviation from zero indicates asymmetry, where positive indicates the slow stance phase had more change from slow baseline condition than fast stance phase. L = left, R = right.

We observed statistically significant associations between gray matter volume and CoM asymmetry. Greater CoM asymmetry was significantly associated with greater gray matter volumes in the brain areas shown in red in Fig. 1A. To reiterate, greater asymmetry in the context of this analysis refers to the asymmetry at the plateau of adaptation during the split-belt walking paradigm (suggestive of more flexible adaptation). The cluster overlaps with the left superior frontal gyrus (*p*$_{FWE-corr}<$ 0.05; Table 2). Figure 1B depicts a scatterplot between the signal from the peak voxel of the gray matter cluster and the behavioral data (CoM asymmetry), shown to illustrate the direction of the relationship.

**Figure 2:**
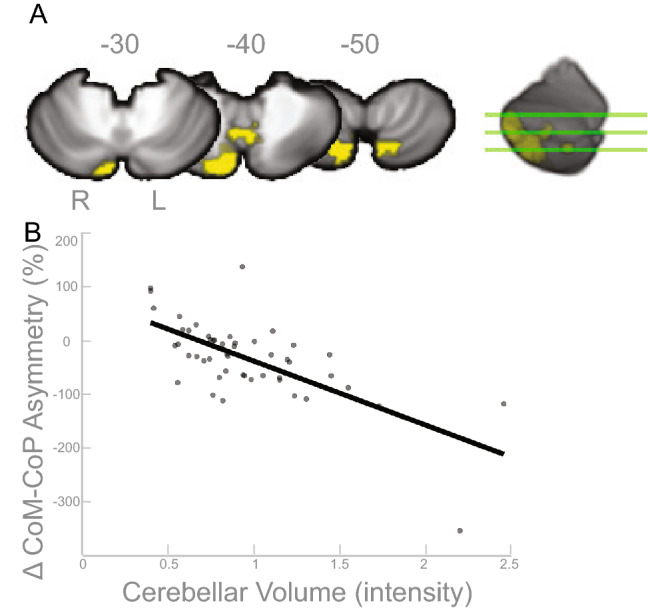
Cerebellar Volume and CoM-CoP Results. (**A**) Results of the FWE corrected t-test looking for clusters in the cerebellar volume that are associated with asymmetry in the CoM-CoP during the split condition of the split-belt treadmill paradigm. The highlighted area corresponds to the cluster/s that passed the *p*$_{FWE-corr}<$ 0.05 threshold. According to the SUIT template, the peaks of the three clusters are located in the right cerebellum lobules VIIB and VIII. (**B**) For illustrative purposes only, the linear relationship of the cerebellar volume at the peak coordinate and asymmetry score are displayed (higher intensity suggests higher volume). The asymmetry score of the gait parameter is calculated as the difference of the variable between slow stance phase and fast stance phase % change from baseline slow condition ($\Delta$). Any deviation from zero indicates asymmetry, where positive indicates the slow stance phase had more change from slow baseline condition than fast stance phase. L = left, R = right.

Additionally, we observed statistically significant associations between cerebellum regional volumes and CoM-CoP. Greater CoM-CoP asymmetry was significantly associated with greater gray matter volumes in the cerebellar areas shown in red in Fig. 2A. The clusters overlap with lobules VIIB and VIII (*p*$_{FWE-corr}<$ 0.05; Table 3). Figure 2B depicts a scatterplot between the peak voxel of the largest cluster and the behavioral data (CoM-CoP), shown to illustrate the direction of the relationship.

**Table 2:** Cerebrum gray matter and CoM results (*p*$_{FWE-corr}<$ 0.05).

Cluster-level			MNI coordinates		
AAL3 label	$K_E$	$P_{FWEcorr}$	x	y	z
Left sup. frontal gyrus	420	0.0164	−15	14	56

Cerebrum volume peak coordinates that have a significant relationship with asymmetry of the CoM during the *split* condition of the split belt treadmill paradigm.

**Table 3:** Cerebellum volume and CoM results (*p*$_{FWE-corr}<$ 0.05).

Cluster-level			MNI Coordinates		
SUIT label	$K_E$	$P_{FWEcorr}$	x	y	z
right Lobule VIIB	6751	0.0014	14	−76	−49
right Lobule VIII	234	0.0386	40	−55	−49
right Lobule VIII	6	0.0492	8	−67	−51

Cerebellum volume peak coordinates that have a significant relationship with asymmetry of the CoM-CoP during the *split* condition of the split belt treadmill paradigm.

### Associations between surface measures and asymmetry

**Figure 3:**
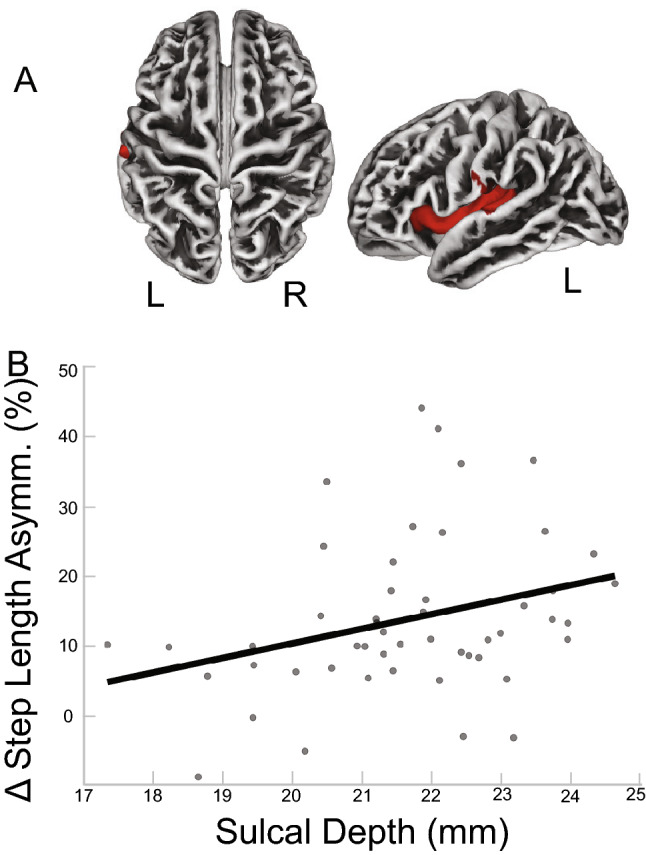
Sulcal Depth and Step Length Results. (**A**) Results of the FWE corrected t-test looking for clusters in sulcal depth that are associated with asymmetry in step length during the split condition of the split-belt treadmill paradigm. The highlighted area corresponds to the cluster/s that passed the *p*$_{FWE-corr}<$ 0.05 threshold. According to the DK40 template, the cluster spans the left insula, superior temporal area, transverse temporal area, postcentral gyrus. (**B**) For illustrative purposes only, the linear relationship of the peak coordinate and asymmetry score are displayed. The asymmetry score of the gait parameter is calculated as the difference of the variable between slow stance phase and fast stance phase % change from baseline slow condition ($\Delta$). Any deviation from zero indicates asymmetry, where positive indicates the slow stance phase had more change from slow baseline condition than fast stance phase. L = left, R = right.

We observed significant associations between sulcal depth and step length. Greater asymmetry in step length was significantly associated with greater sulcal depth in cortical areas shown in red in Fig. 3A. The cluster mostly (50%) overlaps with the left insula, and partially (15,13,12%) with the superior temporal, transverse temporal and postcentral gyri (*p*$_{FWE-corr}<$ 0.05; Table 4). Figure 3B depicts a scatterplot between the peak voxel of the largest cluster and the behavioral data (step length), shown to illustrate the direction of the relationship.

**Table 4:** Sulcal depth and step length results (*p*$_{FWE-corr}<$ 0.05).

DK40 label	Overlap of atlas region (%)	Cluster-level	
		$K_E$	$P_{FWEcorr}$
Left insula	50	1435	0.02900
Left superior temporal	15		
Left transverse temporal	13		
Left postcentral	12		

Regions that overlap with Desikan-Killiany DK40 atlas by 5% or more with the cluster that showed a relationship between sulcal depth and step length asymmetry during the *split* condition of the split belt treadmill paradigm.

**Figure 4:**
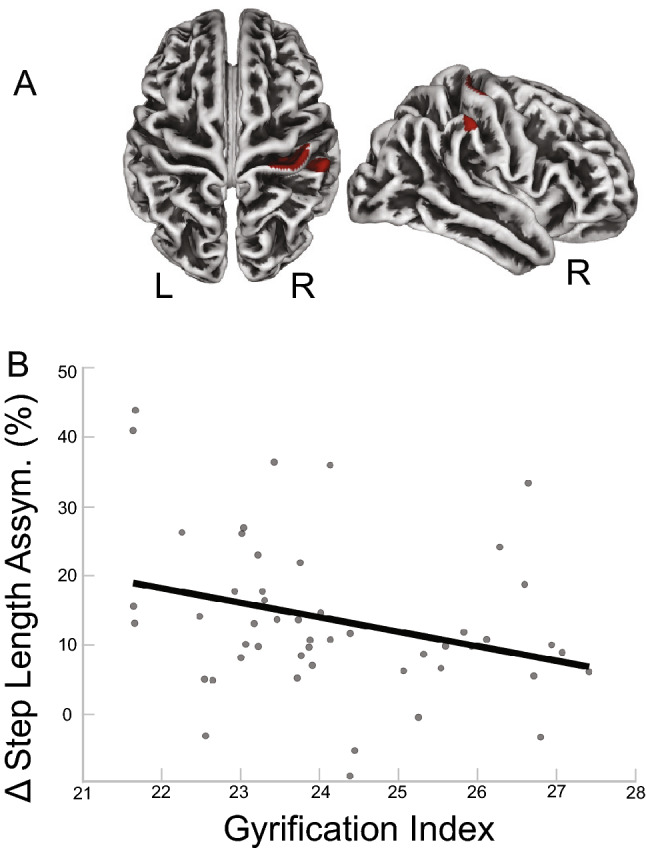
Gyrification and Step Length Results. (**A**) Results of the FWE corrected t-test looking for clusters in gyrification that are associated with asymmetry in step length during the split condition of the split-belt treadmill paradigm. The highlighted area corresponds to the cluster/s that passed the *p*$_{FWE-corr}<$ 0.05 threshold. According to the DK40 template, the clusters span the postcentral, precentral and supramarginal gyri. (**B**) For illustrative purposes only, the linear relationship of the peak coordinate and asymmetry score are displayed. The asymmetry score of the gait parameter is calculated as the difference of the variable between slow stance phase and fast stance phase % change from baseline slow condition ($\Delta$). Any deviation from zero indicates asymmetry, where positive indicates the slow stance phase had more change from slow baseline condition than fast stance phase. L = left, R = right.

**Figure 5:**
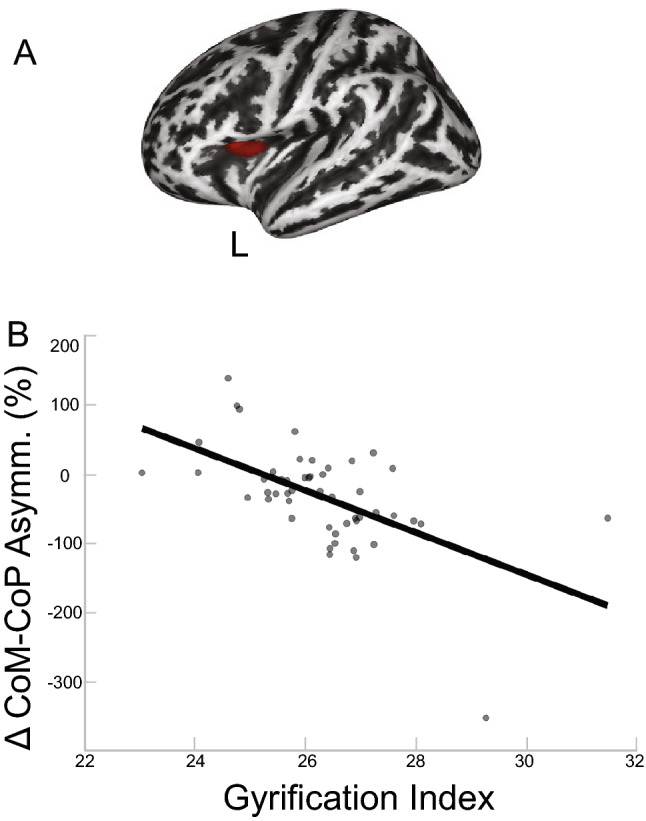
Gyrification and CoM-CoP Results. (**A**) Results of the FWE corrected t-test looking for clusters in gyrification that are associated with asymmetry in CoM-CoP during the split condition of the split-belt treadmill paradigm. The highlighted area corresponds to the cluster/s that passed the *p*$_{FWE-corr}<$ 0.05 threshold. According to the DK40 template, the clusters span the pars opercuralis and insula. (**B**) For illustrative purposes only, the linear relationship of the peak coordinate and asymmetry score are displayed. The asymmetry score of the gait parameter is calculated as the difference of the variable between slow stance phase and fast stance phase % change from baseline slow condition ($\Delta$). Any deviation from zero indicates asymmetry, where positive indicates the slow stance phase had more change from slow baseline condition than fast stance phase. L = left, R = right.

We also observed significant associations between gyrification and asymmetry of two gait parameters. Less asymmetry in step length was significantly associated with greater gyrification in cortical areas shown in red in Fig. 4A. The cluster overlaps with the pre/postcentral gyrus (10 and 90%, respectively) and a smaller cluster overlapped with the supramarginal gyrus (*p*$_{FWE-corr}<$ 0.05; Table 5). Figure 5B depicts a scatterplot between the peak voxel of the poscentral gyrus cluster and the behavioral data (step length), which illustrates the direction of the relationship. We observed another cluster within the gyrification map that showed significant association with asymmetry in CoM-CoP. This result indicates greater CoM-CoP asymmetry is associated with greater gyrification for the brain areas shown in red in Fig. 5A. The cluster overlaps with pars opercularis (67%) and the insula (30%) (*p*$_{FWE-corr}<$ 0.05; Table 6). Figure 5B depicts the scatterplot between the peak voxel of this cluster and the behavioral data (CoM-CoP).

There were no significant associations between cortical thickness or fractal dimension and any gait parameter asymmetry scores.

**Table 5:** Gyrification and step length results (*p*$_{FWE-corr}<$ 0.05). Regions that overlap with Desikan–Killiany DK40 atlas by 5% or more with the cluster that showed a relationship between gyrification and step length asymmetry during the *split* condition of the split belt treadmill paradigm.

DK40 label	Overlap of atlas region (%)	Cluster-level	
		$K_E$	$P_{FWEcorr}$
Right postcentral	90	305	0.0396
Right precentral	10		
Right supramarginal	100	126	0.0398

**Table 6:** Gyrification and CoM-CoP results (*p*$_{FWE-corr}<$ 0.05).

DK40 label	Overlap of atlas region (%)	Cluster-level	
		$K_E$	$P_{FWEcorr}$
Left pars opercularis	67	170	0.033
Left insula	30		

Regions that overlap with Desikan-Killiany DK40 atlas by 5% or more with the cluster that showed a relationship between gyrification and CoM-CoP asymmetry during the *split* condition of the split belt treadmill paradigm.

### Associations between white matter structure (fractional anisotropy) and asymmetry

**Figure 6:**
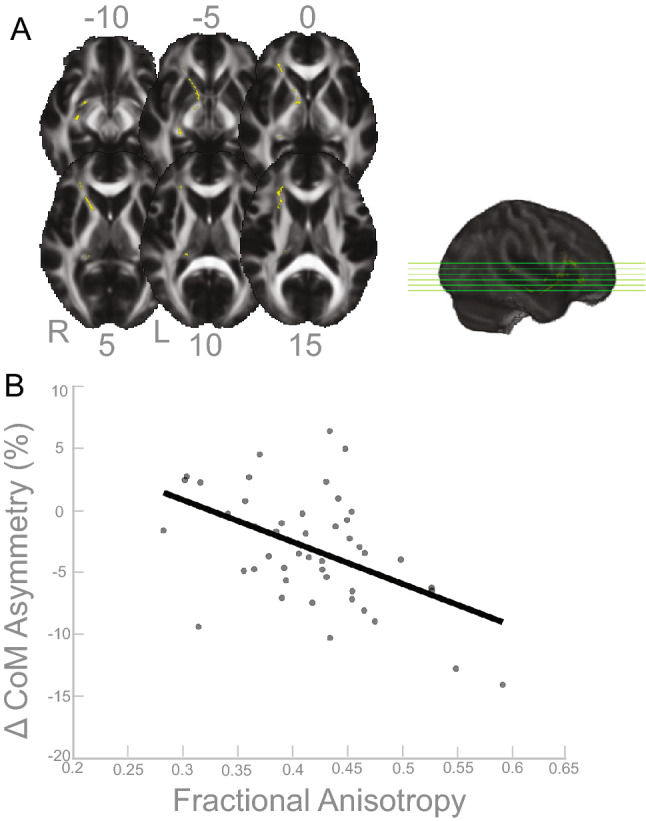
Fractional anisotropy and CoM results. (**A**) Results of the FWE corrected t-test looking for clusters in gyrification that are associated with asymmetry in CoM during the split condition of the split-belt treadmill paradigm. The highlighted area corresponds to the cluster/s that passed the *p*$_{FWE-corr}<$ 0.05 threshold. According to the JHU-ICBM tract atlas, the clusters span the right corticospinal tract and right inferior longitudinal fasciculus. (**B**) For illustrative purposes only, the linear relationship of the peak coordinate and asymmetry score are displayed. The asymmetry score of the gait parameter is calculated as the difference of the variable between slow stance phase and fast stance phase % change from baseline slow condition ($\Delta$). Any deviation from zero indicates asymmetry, where positive indicates the slow stance phase had more change from slow baseline condition than fast stance phase. L = left, R = right.

We observed significant associations between white matter tract microstructure (free water corrected fractional anisotropy) and CoM asymmetry. Greater asymmetry was associated with lower fractional anistropy in the white matter tracts in yellow in Fig. 6A. The clusters overlap with the right corticospinal tract and right inferior longitudinal fasciculus (*p*$_{FWE-corr}<$ 0.05; Table 7). Figure 6B depicts a scatterplot between the peak voxel of the larger corticospinal tract cluster and the behavioral data (CoM), shown to illustrate the direction of the relationship.

**Table 7:** White matter and CoM results (*p*$_{FWE-corr}<$ 0.05).

Cluster-level			MNI coordinates		
JHU-ICBM tract label	$K_E$	$P_{FWEcorr}$	x	y	z
Right corticospinal tract	527	0.025	16	17	−6
Right inf. longitudinal fasciculus	179	0.044	29	24	16
Right corticospinal tract	75	0.046	21	−30	11
Right inf. longitudinal fasciculus	115	0.047	27	33	1

Regions that overlap with JHU-ICBM tract label atlas by 5% or more with the cluster that showed a relationship between FA and CoM asymmetry during the *split* condition of the split belt treadmill paradigm.

### Age group differences in correlations with asymmetry

**Figure 7:**
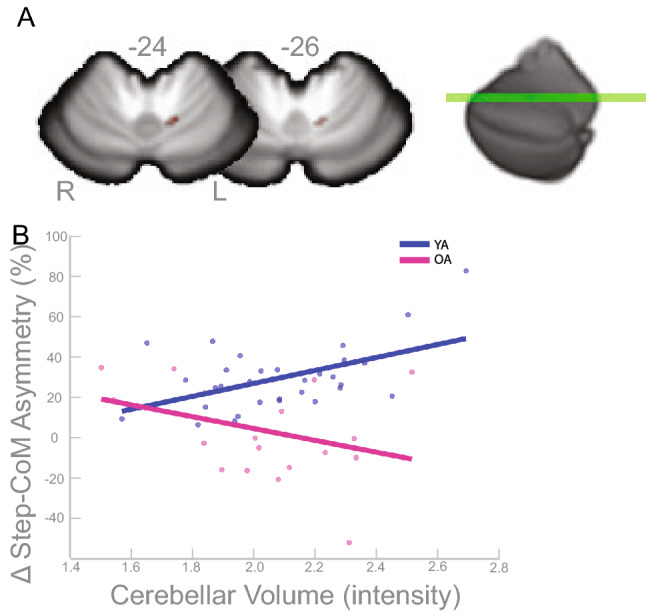
Group Differences in Cerebellum Volume and Step-CoM Results. (**A**) Results of the FWE corrected t-test looking for clusters that show different associations between cerebellar volume and Step-CoM during the split condition of the split-belt treadmill paradigm. The highlighted area corresponds to the cluster/s that passed the *p*$_{FWE-corr}<$ 0.05 threshold. According to the SUIT atlas, the cluster is within left lobule V. (**B**) For illustrative purposes only, the linear relationship of the peak coordinate and asymmetry score are displayed (higher intensity indicates higher volume). The asymmetry score of the gait parameter is calculated as the difference of the variable between slow stance phase and fast stance phase % change from baseline slow condition ($\Delta$). Any deviation from zero indicates asymmetry, where positive indicates the slow stance phase had more change from slow baseline condition than fast stance phase. L = left, R = right.

We observed an association between cerebellar volume and step-CoM, that differed between groups. Specifically, younger adults showed greater asymmetry in step-CoM associated with cerebellar volume within the cluster identified in Fig. 7A, whereas the older adults show the opposite association. The cluster overlaps with cerebellar lobule V (*p*$_{FWE-corr}<$ 0.05; Table 8). Figure 7B depicts a scatterplot between the peak voxel and the behavioral data (step-CoM), shown to illustrate the direction of the relationship for each group.

**Table 8:** Group Differences in Cerebellum Volume and Step-CoM Results (*p*$_{FWE-corr}<$ 0.05).

Cluster-level			MNI Coordinates		
SUIT label	$K_E$	$P_{FWEcorr}$	x	y	z
left V	37	0.0390	−12	−59	−25

Cerebellum volume peak coordinates that have a significant relationship with asymmetry of the Step-CoM during the *split* condition of the split belt treadmill paradigm.

## Discussion

We sought to determine (1) whether any structural brain metrics showed an association with the asymmetry of gait parameters that develops particularly in younger but not older adults during split-belt treadmill adaptation; and (2) whether there were any different brain-behavior relationships between age groups. We identified several brain structural metrics that showed an association with asymmetry, but only one cluster in the cerebellum that had differential associations between age groups. Specifically, the results showed that regardless of age group, greater region specific cerebrum gray matter, cerebellum volume, free-water corrected white matter fractional anisotropy, and cortical gyrification were associated with more asymmetry in ML gait parameters. Region specific sulcal depth and gyrification were associated with step length, and a small cluster in the cerebellum had different brain-behavior relationships with Step-CoM, between groups. Our hypothesis that these associations would be in sensorimotor, cerebellar, and striatal regions was largely supported, although a few associations were also observed for brain regions outside of these areas.

To reiterate the previously reported behavioral results, the AP and ML gait parameters showed different adaptation effects in response to the split-belt treadmill paradigm, in addition to age differences in ML effects of split-belt walking^9^. Specifically, we showed that younger adults adopted more asymmetry in ML gait parameters during split-belt treadmill adaptation, whereas older adults did not adopt this asymmetry. Based on our current understanding of the dynamics involved during human locomotion, and the constraints imposed by the split-belt treadmill paradigm, we believe that the asymmetry adopted by younger adults is metabolically more efficient as they use the passive dynamics of the treadmill belt to their advantage. In addition to these results, multiple reports suggest ML gait parameters require higher level neural control^30–32^, therefore we hypothesized that we would find brain-behavior associations for ML gait parameter asymmetry during split-belt treadmill adaptation, but not AP gait parameters (step length).

The gray matter results indicated that more gray matter in the left superior frontal gyrus was associated with more CoM asymmetry (Table 2 and Fig. 1), and more gray matter in the cerebellar right lobule VIIB and VIII are associated with more CoM-CoP asymmetry (Table 2 and Fig. 2). The superior frontal gyrus is most commonly associated with working memory^57^, but more recent mobile imaging studies suggest it also has a role in walking^58,59^. The gyrification results that involved the ML gait parameters indicated more gyrification in the left pars opercularis/insula was associated with more CoM-CoP asymmetry (Fig. 5). Pars opercularis has a role in imitation and observation of motor tasks, and may contribute to relaying the efferent motor command that predicts future sensory consequences of planned movements^60^. The insula is critical for the function of body awareness and interoception, and links a variety of functions including pain and emotions with our intended actions^61,62^, and particularly the amplitude of movement^63^. The identified functional roles of these brain areas make it conceivable that these regions have important roles in a task that requires great body awareness to take advantage of the passive dynamics of the split-belt treadmill. The fractional anisotropy clusters in the right corticospinal tract and right inferior longitudinal fasciculus showed an association with more CoM asymmetry (Fig. 6). The corticospinal tract and longitudinal fasciculus are major white matter bundles that relay information from cortical areas to the spinal cord and between cortices, respectively^64,65^. All together, the results suggest that regardless of age, those with more gray matter volume, more gyrification, and higher fractional anisotropy in the above mentioned areas were able to adopt this ML asymmetry during the split-belt treadmill paradigm, which is likely a more flexible and efficient control of locomotion. The sulcal depth cluster that showed a significant association with step length, in the left insula, indicated higher sulcal depth was associated with more asymmetry in step length (Fig. 3). The gyrification cluster in the pre/postcentral gyrus showed the opposite relationship with step length (Fig. 4), where increasing gyrification was associated with less asymmetry in step length. Since sulcal depth is generally larger in younger adults^66^ and gyrification is higher in younger adults^67^, the current results might seem to suggest conflicting findings for step length. That is, the healthier brain metric (more sulcal depth and more gyrification) had differing relationships with step length asymmetry. We further discuss the implications and considerations of these findings in the Considerations section.

Interestingly, in most of the statistically significant brain-behavior clusters identified, the peak of the clusters correspond to the input and output of the same side of the body given that the cerebrum has descending contralateral projections^68^ and the cerebellum has descending ipsilateral projections^69^. That is, the clusters that had significant associations with ML gait parameters, namely the cerebrum gray matter cluster (Fig. 1) and the gyrification cluster (Fig. 5) were on the left side of the brain, and the cerebellum cluster was on the right side of the brain (Fig. 2). These locations correspond to control of the right side of the body, which was the side where the stance foot was moving slow (0.7 m/s) during the split condition of the split-belt treadmill paradigm. In the original publication of this behavioral data^9^, we showed that the younger adults, on average, shift their CoM closer to the right side (slow stance foot). We were unable to test the specific biomechanical mechanism that enabled this shift due to data collection limitations, but based on our current understanding we believe that this shift requires additional activation of hip or trunk musculature. The current brain-behavior results may suggest that the slow (right) leg hip musculature is the hip that is being “controlled” to enable the ML asymmetry. Others have shown that stance leg hip proprioception affects ML balance^70–72^.

For our second aim we hypothesized that older adults would have stronger brain-behavior relationships for ML gait parameters, and in different regions relative to young adults, but we only identified one cluster that showed different associations with Step-CoM asymmetry (Fig. 7). The data suggests that only for the young adults, more gray matter in the left cerebellar lobule V was associated with more asymmetry. Lobule V of the cerebellum is known to contribute to encoding movement dynamics^73,74^, and is typically activated during visuomotor adaptation^75–78^. The younger adults adopted more asymmetry, on average, which is in line with the current results that suggest a positive association in lobule V gray matter for the younger adults. The relatively minimal results supporting this hypothesis may be due to the fact that we only examined brain structure, and not also brain function. Our hypothesis was rooted in the theory that older adults compensate for reductions in peripheral and central degradation by shifting neural resources to, what are typically, non-task relevant brain regions^1,79^. However, these theories were based on functional brain measurements, not structural. Future work should investigate the link between brain structure, function, and the relationship with asymmetry of the ML gait parameters during the split condition of split-belt treadmill paradigm.

## Considerations

There are several considerations to keep in mind about this work. First, this is a cross-sectional study that sought to identify (1) structural brain metrics that associated with asymmetry of gait parameters; (2) structural brain metrics that differed between age groups. The relatively small sample size may have limited our ability to detect significant age group differences. Second, it is currently not clear why there would be brain-behavior associations for one ML gait parameter, and not another. Our original behavioral results described in Fettrow et al^11^ were based on group averages, so it is possible that there are different strategies employed by individuals that were not observed in group averages. Additionally, the behavioral variables are represented as percent change to improve the ability to compare between behavioral variables. Still, a percent change in one variable may not have the same behavioral effects as a percent change in another variable. These reasons might explain why some ML variables show associations with brain metrics, but not others. Third, we did not expect step length to have any associations with the brain metrics, but we did observe two results. However, these results conflicted with one another in that the healthier brain metric was associated with more asymmetry (sulcal depth) in one result, and less asymmetry (gyrification) in the other. Gyrification is relatively complex to interpret, for example lower and higher gyrifcation values have been associated with improved cognitive abilities in people with Parkinson’s^80^.

### Conclusion

In conclusion, we identified multiple brain metric clusters that are associated with the development of gait asymmetry during split-belt treadmill adaptation. For all of the significant brain-behavior associations that involved the ML gait parameters, more “healthy” brain metrics (i.e., greater volume, fractional anisotropy, etc) corresponded to more asymmetry. The two significant brain-behavior associations that involved the AP gait parameter showed mixed relationships. This suggests that those who maintain healthy brain structure maintain a more flexible gait pattern pertaining to ML balance throughout their lifespan, and may impact the ability to adapt new locomotion patterns. It may also suggest that step length alterations may not be as influenced by structural brain changes as the ML balance parameters. This work progresses our understanding of how brain structure influences behavior, and in particular balance during walking.

## Data Availability

The datasets and analysis code used during the current study available from the corresponding author on reasonable request.
